# The effectiveness of sertraline in alleviating uremic pruritus in hemodialysis patients: a randomized clinical trial

**DOI:** 10.1186/s12882-023-03212-3

**Published:** 2023-06-03

**Authors:** Mohamed Mamdouh Elsayed, Iman Ezzat Elgohary, Heidi Hesham Said Abdelhamid, Sherif Aziz Zaki

**Affiliations:** 1 Nephrology and Internal Medicine Department, Faculty of Medicine, Alexandria University, Alkhartoom Square, El Azareeta, Alexandria, 21131 Egypt; 2 Internal Medicine Department, Sharq El Madina Hospital, Ministry of Health, Alexandria, Egypt

**Keywords:** Sertraline, Uremic pruritus, Hemodialysis

## Abstract

**Introduction:**

Uremic pruritus (UP) is a common and distressing symptom in end stage renal disease (ESRD) patients. Many approaches have been tested to improve UP without a clear success. We aimed to assess the effect of sertraline on UP in hemodialysis (HD) patients.

**Methods:**

This research is a double-blinded, placebo-controlled, multicentric randomized clinical trial which included sixty patients maintained on regular HD. Patients were allocated to receive sertraline 50 mg twice daily or placebo for 8 weeks. The Visual analogue scale (VAS) and the 5-D itch scale were used to assess pruritus before and after the course of treatment.

**Results:**

At study end in sertraline group, there was a significant decrease from baseline findings in the VAS score (*p* < 0.001), and the 5-D itch scale (*p* < 0.001). On the other hand, in placebo group the VAS score showed a slight non-significant decrease (*p* = 0.469), and the 5-D scale (*p* = 0.584) increased from baseline measurements. The percentage of patients with severe and very severe pruritus decreased significantly in the sertraline group in both scores [(VAS score: *p* = 0.004), (5-D itch score: *p* = 0.002)] with no significant change in the placebo group [(VAS score: *p* = 0.739), (5-D itch scale: *p* = 0.763)]. There was a significant positive relation between the VAS and 5-D itch scores and serum urea with *p* value of 0.002 and 0.001 respectively, and serum ferritin with *p* value of < 0.001 with both.

**Conclusions:**

Patients treated with sertraline had a significant improvement in pruritus as compared with those who received placebo suggesting a potential role for sertraline to treat uremic pruritus in HD patients. Larger randomized clinical trials are needed to confirm these findings.

**Trial registration:**

ClinicalTrials.gov NCT05341843. First registration date: 22/04/2022.

## Introduction

Uremic pruritus (UP) is considered one of the most prevalent and annoying symptoms in end stage renal disease (ESRD) patients [1]. The prevalence of severe UP in hemodialysis (HD) patients has declined from 28% in 1996 to 18% in 2015, however it is still underestimated [2]. Recent studies have linked UP to a poor quality of life (QOL), depression, disturbed sleep, and increased mortality [3–5]. Many factors have been incriminated in the pathogenesis including Th1/Th2 lymphocyte dysregulation, high levels of pro-inflammatory cytokines, increased mast cell activity, xerosis, neuropathy, dysregulation of the endogenous opioid system, anemia, uremic toxins, inadequate dialysis dose, imbalance of calcium, phosphate, and parathormone (PTH) [6–9].

Many therapeutic interventions have been studied to improve UP with variable degrees of success including emollients (improve xerosis), optimizing dialysis (remove uremic toxins), correcting Ca-Ph-parathormone abnormalities, gabapentin and pregabalin (modulating neuropathic pain), montelukast (leukotriene inhibition), naltrexone and nalfurafine (targeting µ and κ receptors), mirtazapine (antagonist of 5HT2 and 5HT3 receptors), difelikefalin (agonist of the κ-opioid receptor) and phototherapy [9–14].

Selective serotonin reuptake inhibitors (SSRIs) have been shown to lower the severity of pruritus [15]. Sertraline hydrochloride is a SSRI which improved itching in patients with cholestatic pruritus [16]. Compared to other SSRI, sertraline is considered a safe and effective option for the treatment of depression and anxiety in ESRD patients [17]. There is paucity of data regarding effects of sertraline on UP. But some have found encouraging results with its use [18]. In this study, we aimed to identify the effect of sertraline in alleviating UP in HD patients.

## Patients and methods

### Study

This research is a double-blinded, placebo-controlled, multicentric randomized clinical trial which enrolled sixty patients from the dialysis units in Alexandria. We included ESRD patients with mild, moderate or severe pruritus who had been assigned to regular long-term HD (thrice-weekly, four-hour HD sessions for more than 30 days), aged 18–80 years. Patients were randomly assigned using block randomization method to receive sertraline 50 mg twice daily or placebo for 8 weeks. Participants, health care providers as well as the outcome assessor were unaware about the type of treatment each patient received. Allocation concealment was ensured using sealed closed envelop randomization technique and every patient was given an identification code. We excluded patients with eczema, psoriasis, allergic dermatitis, drug rash, peripheral neuropathy, thyroid disease, leukemia, lymphoma, liver disease, systemic lupus erythematosus, pregnancy, calcium X phosphorus (Ca X Ph) > 55.0 mg/dl, Ph > 5.5 mg/dl, parathyroid hormone (PTH) > 450 pg/ml, and SSRIs intolerance. We also excluded those who consumed antihistamines, opioid antagonists, immunosuppressants, cholestyramine, corticosteroids, ultraviolet B phototherapy or emollients cream 1 month before study. The trial was registered on Clinicaltrials.gov (NCT05341843) (22/04/2022).

### Methods

All patients were subjected to full history taking with emphases on demographic data, the cause of ESRD, comorbid conditions, the vintage of HD and drug history. Thorough physical examination with a special focus on cutaneous examination for the presence of any scratch marks, excoriation, or signs of skin irritation. Laboratory investigations included complete blood count, serum phosphorus, serum calcium, serum PTH, serum sodium, serum potassium, serum creatinine, blood urea, serum albumin, serum triglycerides, ALT, AST, total bilirubin.

### Pruritus assessment & study outcomes

Primary outcome measure was a change in the UP intensity. The Visual analogue scale (VAS) and the 5-D itch scale were used to assess pruritus before and after the course of treatment (8 weeks). The VAS is a scale consisting of a 10 cm long line and a single question. It is commonly used for measuring itch intensity with high reliability and validity [19]. Scores are based on self-reported measures of symptoms. The VAS has five categories of severity, 0 (no pruritis), < 3 (mild), ≥ 3—< 7 (moderate), ≥ 7—< 9 (severe), ≥ 9 (very severe). The 5-D itch questionnaire was specifically developed to be a measure of itch that is brief, easy to complete and score, sensitive to its effect on quality of life, and capable of detecting change over time. The 5-D itch scale assesses five dimensions of itch (degree, duration, direction, disability, and distribution). Scores range from 5 to 25, with higher scores indicating worse pruritus [20]. Patients evaluated and recorded pruritus using both scores at the beginning of the study and then every 2 weeks till the end.

### Statistical analysis

Data were fed to the computer and analyzed using IBM SPSS software package version 20.0. (Armonk, NY: IBM Corp). Categorical data were represented as numbers and percentages. Chi-square test was applied to investigate the association between the categorical variables. For continuous data, they were tested for normality by the Shapiro–Wilk test. Distributed data were expressed as mean and standard deviation. Student t-test was used to compare two groups for normally distributed quantitative variables while Paired t-test was used to compare between two periods. On the other hand, Mann Whitney test was used to compare two groups for abnormally distributed quantitative variables. Significance of the obtained results was judged at the 5% level. Sample size was calculated using Power Analysis and Sample Size Software (PASS 2020) “NCSS, LLC. Kaysville, Utah, USA, ncss.com/software/pass”. A minimal total hypothesized sample size of 60 eligible hemodialysis patients (30 per group) was needed to assess the effect of sertraline on uremic pruritis in patients undergoing regular HD; taking into consideration 5% level of significance, effect size of 30% and 80% power using Chi Square-test.

## Results

### Patients

**Fig. 1:**
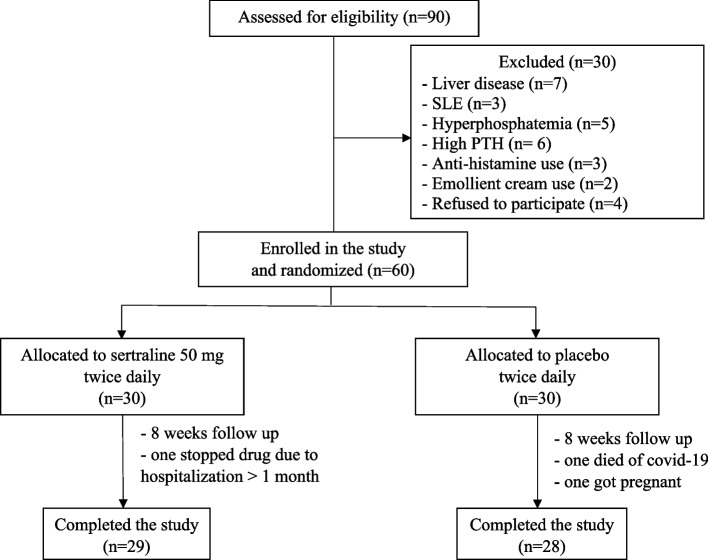
Patient flow chart

The study included a total of 60 HD patients. Following randomization, 30 patients were given 50 mg of sertraline twice daily, while the other 30 received a placebo for an 8-week period. There were 3 dropouts during the study course (Fig. 1). Table 1 displays the clinical features of the patients. Age, sex, body mass index (BMI), cause of ESRD, comorbidities, pruritus duration, duration of HD, vascular access, dialyzer type, dialysis adequacy (Kt/V), and different laboratory parameters did not statistically differ between the two groups.

**Table 1:** Baseline characteristics of the study groups

	**Sertraline group (** ***n*** ** = 30)**	**Placebo group (** ***n*** ** = 30)**	***P*** ** value**
**Age** (years)	43.67 ± 12.63	50.27 ± 13.09	0.052
**Sex** (No)			
Male	10 (33.3%)	13 (43.3%)	0.426
Female	20 (66.7%)	17 (56.7%)	
**BMI** (kg/m^2^)	26.02 ± 5.80	25.74 ± 4.42	0.833
**Pruritis duration** (years)	1.70 ± 1.24	1.92 ± 1.50	0.764
**Duration of HD** (years)	4.69 ± 4.34	4.12 ± 3.38	0.673
**Cause of ESRD** (No)			
-Hypertension	14	16	
-DM	7	3	
-APCKD	2	1	
-GN	5	9	
-Others	2	1	
**Vascular access** (No)			
-AV fistula	25 (83.3%)	27 (90%)	0.706
-Tunneled catheter	5 (16.7%)	3 (10%)	
**Dialyzer type** (No)			
-Low flux	7 (23.3%)	11 (36.7%)	0.260
-High flux	23 (76.7%)	19 (63.3%)	
**Comorbidities** (No)			
-Hypertension	29	29	
-DM	8	6	
-IHD	11	18	
**Phosphate binders** (No)			
-Calcium Based	17	15	
-Non-calcium Based	3	2	
**Alfacalcidol use** (No)	11	9	
**Total cholesterol** (mg/dl)	184.5 ± 53.25	179.1 ± 27.37	0.564
**Serum triglycerides** (mg/dl)	122.07 ± 28.89	126.23 ± 39.95	0.941
**Hemoglobin** (g/dl)	9.98 ± 1.63	9.81 ± 1.63	0.682
**Serum albumin** (g/dl)	4.17 ± 0.39	3.98 ± 0.79	0.614
**SGPT** (u/l)	19.27 ± 7.32	18.37 ± 9.54	0.574
**Total bilirubin** (mg/dl)	0.69 ± 0.20	0.58 ± 0.21	0.064
**Serum calcium** (mg/dl)	8.31 ± 0.95	8.59 ± 0.77	0.210
**Serum phosphorus** (mg/dl)	4.65 ± 0.74	4.57 ± 0.53	0.067
**Ca X P product**	43.12 ± 21.09	38.99 ± 9.22	0.544
**Serum PTH** (pg/ml)	298.85 ± 120.23	234.73 ± 234.73	0.082
**Ferritin** (ng/ml)	512.57 ± 269.35	562.27 ± 266.0	0.455
**Urea** (mg/dl)	148.62 ± 40.20	129.77 ± 36.17	0.061
**Creatinine** (mg/dl)	11.34 ± 2.54	10.11 ± 3.52	0.125
**Kt/V**	1.19 ± 0.08	1.17 ± 0.12	0.683

Data were expressed as mean ± standard deviation (SD), or absolute numbers as appropriate

*APCKD* adult polycystic kidney disease, *BMI* body mass index, *DM* diabetes mellitus, *ESRD* end stage renal disease, *GN* glomerulonephritis, *HD* hemodialysis, *IHD* ischemic heart disease, *Kt/V* measuring dialysis adequacy, *PTH* parathyroid hormone, *SGPT* serum glutamic pyruvic transaminase

### Effect of sertraline and pruritus assessment by VAS and 5-D itch scores

**Fig. 2:**
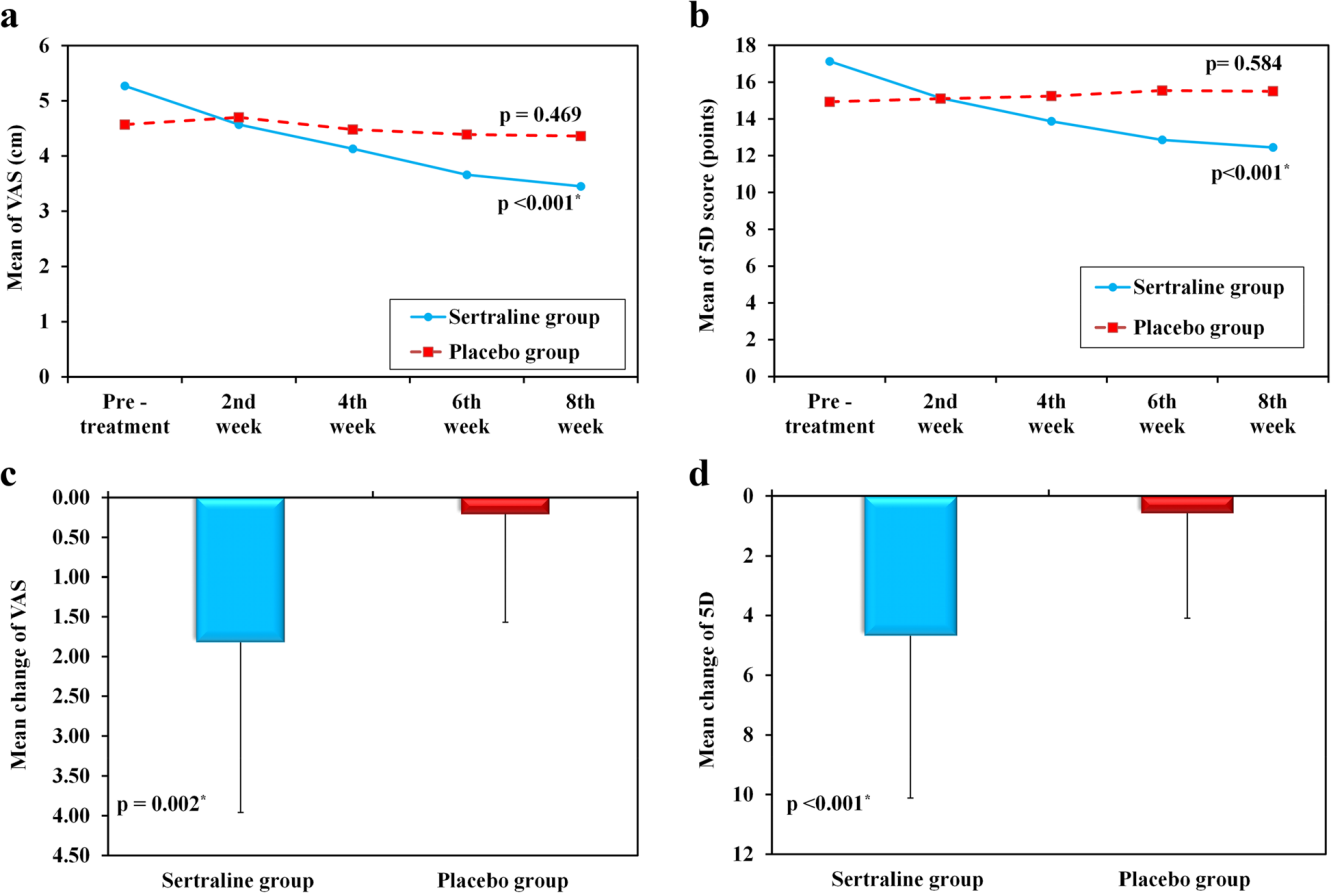
**a** Change in VAS score during study period in each group, **b** Change in 5-D itch scale during study period in each group, **c** Mean change in VAS score at week 8, **d** Mean change in 5-D itch scale at week 8

At baseline, There was no significant difference between the two groups regarding VAS and 5-D itch scores. At the end of the study, the sertraline group's VAS and 5-D scores decreased significantly (*p* < 0.001, in both scores). In placebo group after 8 weeks, the VAS score showed a very slight non-significant decrease (*p* = 0.469) and the 5-D score increased (*p* = 0.584). Furthermore, the VAS and 5-D scores' change (Δ) in sertraline group significantly exceeded that of the placebo group with *p* value of 0.002 and < 0.001, respectively (Table 2) (Fig. 2).

**Table 2:** Pruritus assessment using VAS and 5-D scores at baseline and study end in both groups

	Sertraline group (*n* = 30)				Placebo group (*n* = 30)				Comparison between groups		
	Baseline	Week 8	*p*_*0*_	Change (Δ)	Baseline	Week 8	*p*_*0*_	Change (Δ)	*p*_*1*_	*p*_*2*_	*p*_*3*_
**VAS score** (cm)	5.27 ± 2.75	3.45 ± 2.03	< 0.001	↓1.82	4.57 ± 1.94	4.36 ± 2.34	0.469	↓0.21	0.435	0.175	0.002
**5D itch scale** (point)	17.13 ± 6.99	12.45 ± 3.78	< 0.001	↓4.68	14.93 ± 4.31	15.50 ± 4.81	0.584	↑0.57	0.410	0.009	< 0.001

Data were expressed in Mean ± SD

*p*_*0*_: *p* value for comparing between Baseline and Week 8 in each group

*p*_*1*_: *p* value for comparing between sertraline group and Placebo group at Baseline

*p*_*2*_: *p* value for comparing between sertraline group and Placebo group at Week 8

*p*_*3*_: *p* value for comparing the change (Δ) between sertraline group and Placebo group

VAS: visual analogue scale

**Fig. 3:**
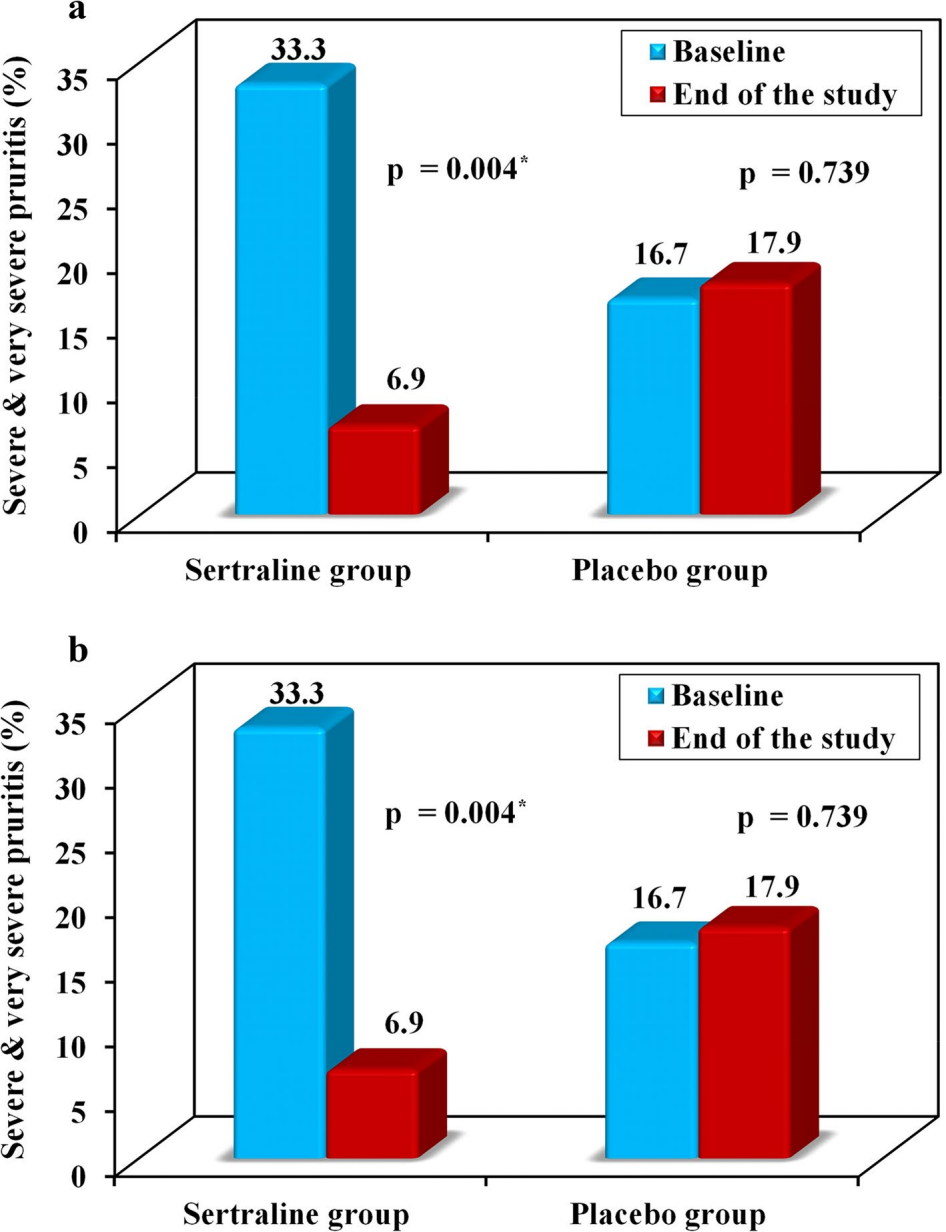
**a** Change in pruritus severity according to VAS score, **b** Change in pruritus severity according to 5-D itch scale

Also, at the end of the study, the percentage of patients with severe and very severe pruritus grade decreased significantly in the sertraline group in both scores [(VAS score: decreased from 33.3% to 6.9%, *p* = 0.004), (5-D itch scale: decreased from 36.7% to 17.2%, *p* = 0.002)]. While in the placebo group after 8 weeks, the percentage of severe and very severe cases revealed no significant change [(VAS score: increased from 16.7% to 17.9%, *p* = 0.739), (5-D itch scale: decreased from 30% to 28.6%, *p* = 0.763)] (Fig. 3).

A summary of the adverse events among the studied patients is shown in Table 3. The most frequent adverse events were diarrhea, nausea, dyspepsia, insomnia, headache, and xerostomia. The incidence rates of adverse events were similar in both groups with no significant difference.

**Table 3:** Adverse events in both groups

Side effects	Sertraline group (*n* = 30)	Placebo group (*n* = 30)	*P* value
**Xerostomia**	3 (10%)	2 (6.7%)	1.000
**Dyspepsia**	5 (16.7%)	7 (23.3%)	0.519
**Nausea**	5 (16.7%)	4 (13.3%)	1.000
**Diarrhea**	4 (13.3%)	1 (3.3%)	0.353
**Insomnia**	3 (10%)	2 (6.7%)	1.000
**Headache**	3 (10%)	0 (0%)	0.237

Data were expressed as absolute numbers (%)

### Relation between VAS and 5-D itch scores with different parameters

At baseline, there was a statistically significant positive relation between the VAS and 5-D scores and serum urea with *p* value of 0.002 and 0.001 respectively. Also, there was a significant positive relation between the VAS and 5-D scores and serum ferritin with *p* value of < 0.001 and < 0.001 respectively. Regarding sex, female patients had higher pruritus scores compared to males and this difference was significant in the VAS score (*p* = 0.009), but not significant in the 5-D itch scale (*p* = 0.593) (Table 4).

**Table 4:** Relation between VAS and 5-D scores with different parameters at baseline

variables	VAS		5-D	
	r_s_	*p*	r_s_	*p*
**HD vintage**	0.094	0.477	0.148	0.258
**Pruritis duration**	0.153	0.243	0.197	0.132
**Hemoglobin**	-0.129	0.327	-0.117	0.372
**WBCs**	0.154	0.239	0.161	0.219
**Platelets**	0.104	0.429	0.077	0.557
**Urea**	0.387	0.002	0.409	0.001
**Creatinine**	-0.171	0.191	-0.236	0.069
**KT/V**	0.097	0.461	0.122	0.353
**Calcium**	-0.192	0.141	-0.241	0.064
**Phosphorus**	-0.229	0.078	-0.185	0.157
**CaXPo4**	-0.226	0.083	-0.216	0.097
**PTH**	0.080	0.545	0.051	0.698
**Ferritin**	0.572	< 0.001	0.534	< 0.001
**Albumin**	-0.120	0.359	-0.123	0.350
**ALT**	-0.035	0.790	0.013	0.922
**AST**	-0.017	0.899	0.023	0.860
**VAS**			0.966	< 0.001

r_s_, Spearman coefficient

## Discussion

Uremic pruritus is a frequent and annoying symptom in hemodialysis patients, and therefore drugs with enhanced efficacy and good tolerability are needed. In the present study, sertraline significantly improved pruritus intensity, as compared with placebo.

The problem with UP is that its pathogenesis is multifactorial and not clear [21]. Also, most of the trials testing UP treatment have been conducted on small populations with inconsistent results making it very difficult to establish a solid guideline for treatment of UP in dialysis patients [22].

Depression affects pruritus patients more frequently, and depression can intensify itching [23]. So, we tried to assess the effects of sertraline in UP as it is considered one of the safest antidepressants in dialysis patients. As illustrated above, with sertraline use we found that pruritus decreased significantly as assessed by VAS, and 5-D itch scores and percentage of patients with severe grades of pruritus decreased significantly. Only few studies have been done to test sertraline in UP. Shakiba et al., enrolled 19 HD patients, graded pruritus by the 30-item inventory of pruritus and found a decrease in pruritus intensity with sertraline use (50 mg/day for 4 months) [24]. Also, Pakfetrat et al., in their study which included 50 HD patients, used VAS and DUO pruritus scoring systems and found similar results (100 mg/day) [18]. Chan et al., in their research (n = 20) reported that sertraline (25–200 mg) was effective in treatment of UP patients who did not respond to antihistamines [25]. There might be potential mechanisms for the beneficial effects we found with sertraline use. Many authors highlighted the role of inflammation in ESRD itch [26, 27]. Sertraline was found to reduce inflammatory cytokines like IL-6 and tumor necrosis factor alpha in ESRD patients [28]. Also, because oral antidepressants affect serotonin and histamine levels, they are believed to have an antipruritic effect. And so, The European Guideline for Chronic Pruritus recommends them for cases where other treatments are ineffective [29].

The efficacy of sertraline in our work (assessed by 5-D itch scale) was comparable to the outcomes of other UP medications such as difelikefalin [30], pregabalin and gabapentin [31].

In the present work, we found a statistically significant relation between pruritus and serum urea. The dialysis adequacy in both groups (represented as Kt/V) was slightly less than the target values in HD patients and this could explain our finding. Similar findings were reported by other researchers [18, 32]. However, these findings contradicted those of Bolanos et al. [33]. Also, we found a statistically significant positive relation between ferritin and pruritus, which is in line with the findings of Virga et al. [34] supporting the role of inflammation in UP. However, other researchers [32, 33] revealed no association. In the current study, we found that female patients had more pruritus compared to males. This is consistent with the findings of Ramakrishnan et al. [35] and Ersoy et al. [36]. On the other hand, other researchers [37, 38] found that male sex is associated more with pruritus. Also, some studies found no relation between sex and UP [39, 40].

We found no association between pruritus and calcium, phosphorus, PTH, serum albumin, and Kt/V. This might be due to our exclusion of patients with hyperphosphatemia, hyperparathyroidism, or high Ca X Ph product from the study. This is consistent with the findings of other studies [18, 33]. However, other studies revealed an association between UP and some of these variables [32, 39, 41].

Regarding sertraline safety, we found no significant difference in the reported adverse events in both groups. Also, no major adverse event, bleeding, new sexual complaint, nor QT interval prolongation were reported in the sertraline group. These findings support the data [17] suggesting that sertraline is considered a relatively safe antidepressant in dialysis patients with fewer side effects compared to other drugs.

The strengths of our study include being one of the largest trials to investigate sertraline effects on UP till now. Also, we used two itch scores to support our findings and the results were consistent in both. A possible drawback of our study might be the follow up period (8 weeks), longer durations would have strengthen our findings. Also, the Kt/V in both groups was slightly less than the required value (1.2) in HD patients.

## Conclusion

Pruritus intensity among HD patients decreased significantly with sertraline use. These results may point to a role for sertraline in the treatment of UP. To support these results, larger, randomized clinical trials with longer follow-up periods are required.

## Data Availability

All data analyzed during this study are included in this manuscript.
